# Glutamic acid decarboxylase 1 alternative splicing isoforms: characterization, expression and quantification in the mouse brain

**DOI:** 10.1186/1471-2202-15-114

**Published:** 2014-10-16

**Authors:** Stefan Trifonov, Yuji Yamashita, Masahiko Kase, Masato Maruyama, Tetsuo Sugimoto

**Affiliations:** Department of Anatomy and Brain Science, Kansai Medical University, Hirakata, Osaka, 573-1010 Japan

**Keywords:** GAD1, GAD2, Alternative splicing, Quantitative RT-PCR, *in situ* hybridization

## Abstract

**Background:**

GABA has important functions in brain plasticity related processes like memory, learning, locomotion and during the development of the nervous system. It is synthesized by the glutamic acid decarboxylase (GAD). There are two isoforms of GAD, GAD1 and GAD2, which are encoded by different genes. During embryonic development the transcription of GAD1 mRNA is regulated by alternative splicing and several alternative transcripts were distinguished in human, mouse and rat. Despite the fact that the structure of GAD1 gene has been extensively studied, knowledge of its exact structural organization, alternative promoter usage and splicing have remained incomplete.

**Results:**

In the present study we report the identification and characterization of novel GAD1 splicing isoforms (GenBank: KM102984, KM102985) by analyzing genomic and mRNA sequence data using bioinformatics, cloning and sequencing. Ten mRNA isoforms are generated from GAD1 gene locus by the combined actions of utilizing different promoters and alternative splicing of the coding exons. Using RT-PCR we found that GAD1 isoforms share similar pattern of expression in different mouse tissues and are expressed early during development. Quantitative RT-PCR was used to investigate the expression of GAD1 isoforms and GAD2 in olfactory bulb, cortex, medial and lateral striatum, hippocampus and cerebellum of adult mouse. Olfactory bulb showed the highest expression of GAD1 transcripts. *Isoforms 1/2* are the most abundant forms. Their expression is significantly higher in the lateral compared to the medial striatum. *Isoforms 3/4*, *5/6*, *7/8* and *9/10* are barely detectable in all investigated regions except of the high expression in olfactory bulb. When comparing GAD1 expression with GAD2 we found that *Isoforms 1/2* are the predominant isoforms. *In situ* hybridization confirmed the predominant expression of *Isoforms 7/8* and *9/10* in the olfactory bulb and revealed their weak expression in hippocampus, cerebellum and some other areas known to express GAD1.

**Conclusions:**

Generation of ten splicing isoforms of GAD1 was described including two so far uncharacterized transcripts. GAD1 splicing isoforms producing the shorter, enzymatically inactive GAD25 protein are expressed at very low level in adult mouse brain except in the olfactory bulb that is associated with neurogenesis and synaptic plasticity even during adulthood.

**Electronic supplementary material:**

The online version of this article (doi:10.1186/1471-2202-15-114) contains supplementary material, which is available to authorized users.

## Background

γ-aminobutyric acid (GABA) is the major inhibitory neurotransmitter in the adult mammalian central nervous system. It is a key element in processes like locomotion, reproduction, learning and is involved as a trophic factor in the proliferation, migration and differentiation of neurons during embryonic development [1, 2]. GABA is synthesized from glutamate by the enzyme glutamate decarboxylase (GAD). GAD exists as two isoforms, GAD1 and GAD2, having different molecular weights of 67 kDa and 65 kDa, respectively. They are encoded by separate genes [3–5] and are coexpressed in most of the GABA-containing neurons, but often in variable ratios [3, 6–9]. GAD isoforms share enormous sequence homology at protein level, but have different affinity to the cofactor pyridoxal 5′-phosphate and distinct intracellular localizations, which suggests that they might be involved in the synthesis of different pools of GABA with distinct functions [1, 4, 7, 10, 11].

Structure, alternative promoters and putative regulatory elements of the mouse GAD1 gene were described previously by Szabo et al. [12] and Yanagawa et al. [13]. The mouse, rat and human GAD1 genes are very similar, with identical size of exons and conserved splice junction sites [12–15]. There are increasing evidences for the existence of transcriptional regulation mechanism for GAD1 gene but not GAD2 gene. During embryonic development and under some physiological and pathophysiological conditions the transcription of GAD1 mRNA is regulated by alternative splicing [16–18]. In the course of mouse and rat embryogenesis, two additional forms of GAD1 were synthetized by alternative splicing [19–21] encoding 25 kDa leading peptide (GAD25), that corresponds to the amino-terminal regulatory region of GAD1 and another enzymatically active form GAD44. In mice and rats the two additional transcripts are distinguished by insertion of either 80 or 86 base pairs (bp) into the full-length GAD1 message upstream of the pyridoxal 5′-phosphate-binding site [19, 20]. These alternatively spliced exons contain an in-frame overlapping stop/start codon, thus both alternative transcripts code for a short enzymatically inactive GAD protein of 25 kDa (GAD25). The shorter 80-bp exon lacks another stop codon at the 3′-end and termination-reinitiation at the stop/start codon produces an enzymatically active protein of 44 kDa (GAD44) corresponding to the carboxyl terminal domain of GAD1, that contains the pyridoxal 5′-phosphate-binding site. Alternative splicing of GAD1 gene has been also described in human pancreatic islet cells, adrenal cortex and testis. Human alternatively spliced mRNA is shorter than the GAD1 mRNA and comprises only of the first seven exons of the human GAD1 gene, thus encoding a shorter 25-kDa protein [22, 23]. In rat testis and other tissues four novel GAD1 mRNA isoforms synthetized by alternative splicing combined with the utilization of an intron located polyadenylation site and additional transcription start site located in Intron3 were recently described [24, 25].

Despite the fact that the structure and regulatory elements of mouse GAD1 gene have received close attention in the past decades, knowledge of the exact structural organization, alternative promoter usage and splicing of the mouse GAD1 gene have remained incomplete. Here, we report the identification and characterization of novel mouse GAD1 mRNA splicing isoforms (GenBank: KM102984, KM102985) in addition to the previously described and introduce a new numbering system for the mouse GAD1 exons. We also report the distinct tissue-specific expression profiles of the GAD1 splicing isoforms and the activity of the different promoters in selected adult mouse brain regions and non-neuronal tissues by sensitive methods like RT-PCR, quantitative RT-PCR, and *in situ* hybridization.

## Methods

### Animals

All experiments were performed in compliance with the National Institutes of Health Guide for the Care and Use of Laboratory Animals (NIH Publication No. 80–23, revised 1996) and the Kansai Medical University local guidelines for animal experimentation (issued 9 March 1999; registration number of the current research proposal, 12–064; permit number, 25–055). This study had full approval from the Institutional Committee for Animal Experimentation. All efforts were made to reduce the number of animals and their suffering.

### *In silico*analysis of the structure of mouse GAD1 gene

Mouse GAD1 gene structure *in silico* analysis was performed using genomic, mRNA and EST databases (http://www.ncbi.nlm.nih.gov/). GAD1 mRNA sequence (GenBank: NM_008077.4) was set to query against the database collections and high homology was found with two mouse mRNA sequences (GenBank: AK047521, AK054554) and one human mRNA sequence (GenBank: NM_013445.3). These sequences were used for homology searches and analysis against GAD1 reference genomic sequence and the sequence of the promoter region (GenBank: NC_000068.7, Z49978). Multiple alignment, homology search and ORF prediction were performed by GENETYX 10.0 (http://www.genetyx.co.jp) and SIM4 (http://pbil.univ-lyon1.fr/members/duret/cours/inserm210604/exercise4/sim4.html) for aligning cDNA and genomic DNA to identify exon and intron regions. Multiple alignment of amino acid sequences predicted from GAD1 splicing isoforms and analysis of interspecies homologies were performed with CLUSTALW2 (http://www.ebi.ac.uk/Tools/clustalw2/index.html).

### Cloning and expression of GAD1 splicing isoforms in different mouse tissues

Total RNA was obtained from the brain of an adult male C57BL/6J mouse as previously described [9, 26, 27]. Briefly, 1 μg of total RNA was used for first-strand cDNA synthesis using 500 ng of oligo(dT) primer (Invitrogen, Life Technologies, Carlsbad, CA, USA), 500 μM dNTPs (Takara, Otsu, Japan), 40 units of ribonuclease inhibitor (Toyobo, Tokyo, Japan), 5 mM DTT and 200 units of SuperScript III (Invitrogen) in 20 μl of reaction mixture for 10 minutes at room temperature to allow primer annealing, and then for 1 hour at 50°C. GAD1 isoforms were amplified using common forward primer in Exon1 (5′-cttcttcaggctctcccgtgc-3′) or Exon2 (5′-tctcctttttaccctctgcca-3′) and a unique reverse primer. *Isoforms 3, 4, 5* and *6* were amplified in two separate parts by using forward (5′-gggagtgttggttgctacggtgatg-3′) and reverse (5′-gtcacactcatgctttggttctgctc-3′) primers in Exon8 in tandem with the common forward and reverse (5′-cagctaagcgagtcacagagattggtca-3′) primers for *Isoforms 1* and *2*. The sequence of the reverse primer used to amplify *Isoforms 7* and *8* was: 5′-cagggtccaagagaagtcaaggaagacc-3′. The reverse primer used to amplify *Isoforms 9* and *10* was: 5′-agccagctaggtgatggtca-3′. All isoforms were cloned in pGEM-T Easy (Promega, Madison, WI) and sequenced with ABI PRISM BigDye Terminator Cycle Sequencing Ready Reaction Kits (Applied Biosystems, Tokyo, Japan).

**Figure 1:**
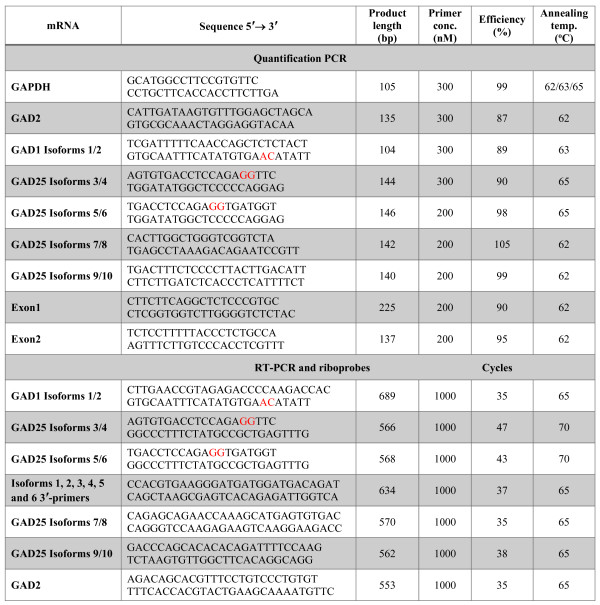
**Sequence of primers used for quantitative RT-PCR, RT-PCR and preparation of riboprobes for** ***in situ*** **hybridization.** Locked nucleic acid substitutions in the primers are denoted with red capital letters.

Mouse Multiple Tissue cDNA (MTC) Panel I (Clontech Laboratories Inc., Mountain View, CA) and cDNAs from pancreas, small intestine and large intestine (GenoStaff, Tokyo, Japan) were used as templates to determine the expression of GAD1 isoforms and GAD2 in different mouse tissues. The primer pairs used in the RT-PCR analysis are listed in Figure 1. The PCR mix (25 μl) contained 2.5 μl template cDNA, 1 μM forward and reverse primers, 200 μM dNTPs (Takara), and 0.5 U Ex Taq HS (Takara). The number of cycles and the annealing temperature differed for each isoform and are listed in Figure 1.

### Quantitative RT-PCR

Total RNA was obtained from five ten week-old, male C57BL/6J mice. They were housed under standard conditions with free access to food and water. The animals were deeply anesthetized with an intraperitoneal injection of sodium pentobarbital (100 mg⁄kg), and the brains were rapidly dissected. The olfactory bulb and the cerebellum were separated primarily. Then coronal sections of 1 mm thickness were dissected from the cerebrum on an adult coronal brain matrix (RBMS-200C, Kent Scientific Corp., USA) between interaural positions +7.0 mm to +3.8 mm according to Paxinos and Franklin [28]. Frontal cortex was taken between cuttings +7.0 mm and +5.5 mm. Medial striatum and lateral striatum were punched out between cuttings +5.2 mm and +3.8 mm. The whole hippocampi were dissected from the rest of the brain. Specimens were collected in RNAlater (Ambion, Life Technologies, Carlsbad CA, USA) and kept at −80°C. Total RNA was purified using RNAqueous-4PCR Kit (Invitrogen) followed by DNase treatment according to manufacturer’s instructions. RNA was spectrophotometrically quantified and only RNA with A_260_/A_280_ ratio between 1.8 and 2.1 were used. 500 ng of total RNA in 20 μl total volume were reverse transcribed with SuperScript VILO cDNA Synthesis Kit (Invitrogen). Resulting cDNAs were stored at −20°C. Primers used for amplification of GAD transcripts and the reference gene GAPDH (glyceraldehyde 3-phosphate dehydrogenase) are listed in Figure 1 and purchased from Invitrogen. To increase the specificity of the primers for *Isoforms 1* to *6* and to improve the discrimination between these highly homologous sequences the critical nucleotides were substituted with locked nucleic acids [29, 30]. Those primers were purchased from GeneDesign (Osaka, Japan). Quantitative RT-PCR was carried out on StepOne Real-Time PCR System (Applied Biosystems) using KOD SYBR qPCR Mix (Toyobo). The following PCR protocol was applied: 40 or 45 cycles of 98°C for 10 sec; 62, 63 or 65°C (primer pair specific; see Figure 1) for 10 sec; 68°C for 30 sec. All samples were run in duplicates and the analysis was performed on StepOne Software 2.3 (Applied Biosystems). External standard curves were used for absolute quantification. gBlock Gene Fragments (Integrated DNA Technologies) were used as standards (Additional file 1) and the number of transcripts were calculated per 10000 transcripts of GAPDH.

All data are shown as mean ± standard error of the mean (SEM) of 5 individual animals. Statistical analysis was performed using GraphPad Prism version 6.00 for Windows (GraphPad Software, San Diego California USA, http://www.graphpad.com). One-way ANOVA followed by Tukey’s post hoc test was used to compare the expression of: (1) each isoform, Exon1 and Exon2 between the various mouse brain regions; (2) different isoforms in one brain region. Differences between Exon1 and Exon2 expression in each of the investigated brain regions was evaluated by Mann–Whitney *U* test. *P* < 0.05 was considered statistically significant.

### Riboprobe synthesis and *in situ*hybridization histochemistry

Total RNA was obtained from an adult male C57BL/6J mouse and the first-strand synthesis was performed as described above for the cloning of GAD1 splicing isoforms. One microliter aliquot of reverse-transcribed product was amplified by PCR using forward and reverse primers listed in Figure 1. All fragments were subcloned into plasmid pGEM-T Easy vector (Promega) and sequenced with ABI PRISM BigDye Terminator Cycle Sequencing Ready Reaction Kits (Applied Biosystems). Purified plasmids were linearized and subjected to in vitro transcription. The reaction was carried out for 2 hours at 37°C in 20 μl of transcription buffer, containing 150 ng template DNA, 1 mM GTP, 1 mM ATP, 1 mM CTP, 0.65 mM UTP, 0.35 mM DIG-11-UTP (DIG RNA Labeling Mix, Roche Diagnostics, Mannheim, Germany), 20 units of ribonuclease inhibitor (Toyobo), and 40 units of T7 (Stratagene, La Jolla, CA) RNA polymerase. Riboprobes were precipitated with ethanol and LiCl, resuspended in diethylpyrocarbonate (DEPC)-treated dH_2_O and spectrophotometrically quantitated.

Three digoxigenin-labelled riboprobes were obtained in antisense and sense strand, namely: (1) Probe-GAD1 – against 3′-region of *Isoforms 1* to *6*; (2) Probe-GAD25-7/8 – against 3′-noncoding region of *Isoforms 7* and *8*; (3) Probe-GAD25-9/10 – against the 3′-noncoding region of *Isoforms 9* and *10.* Specificity of Probe-GAD1 and its pattern of hybridization in adult mouse brain are described in details in a previous report [9] and the probe was used here only for Southern blotting experiment. Free-floating tissue sections from ten week-old male C57BL/6J mice were processed for *in situ* hybridization as described previously [9, 26, 27]. Briefly, tissue sections were rinsed in 0.1% DEPC-activated 0.1 M phosphate-buffered saline (pH 7.4) at room temperature twice for 15 minutes. The sections were then equilibrated in 5 × SSC (20 × SSC corresponds to 0.3 M sodium citrate and 3 M sodium chloride, pH 7.0) for 15 minutes and subjected to prehybridization and hybridization procedures.

Free-floating sections were placed initially into hy-bridization buffer containing 50% deionized formamide, 40 μg/ml denatured salmon sperm DNA and 5 × SSC solution at 55°C for 2 hours. Hybridization was performed in hybridization mixture with approximately 1 μg/ml of each digoxigenin-labelled riboprobe in hybridization buffer at 55°C overnight. Following this reaction, the sections were rinsed with 2 × SSC solution at room temperature for 30 minutes; with 2 × SSC solution at 65°C for 60 minutes; and three times, for 20 minutes each time, in 0.1 × SSC solution at 65°C. Digoxigenin-labelled riboprobes were detected with anti-digoxigenin alkaline phosphatase conjugated Fab fragments (Roche Diagnostics), diluted 1:5000 in DIG buffer 1 (0.1 M Tris hydrochloride, 0.15 M sodium chloride, pH 7.5) containing 0.5% blocking reagent (Roche Diagnostics). The chromogenic reaction was carried out in DIG buffer 2 (0.1 M Tris hydrochloride, 0.1 M sodium chloride, 0.05 M magnesium chloride, pH 9.5) containing 450 μg/ml nitroblue tetrazolium chloride (NBT, Roche Diagnostics) and 175 μg/ml 5-bromo-4-chloro-3-indolyl-phosphate (BCIP, Roche Diagnostics) in light-protected conditions overnight at 37°C. The enzymatic reaction was terminated by rinsing the sections in TE buffer (10 mM Tris hydrochloride, 1 mM EDTA, pH 8.0) for 15 minutes. Finally, tissue sections were washed in 0.9% saline solution, mounted on gelatin-coated slides, dehydrated with successive changes of ethanol (50%, 75%, 95%, 100% and 100% ethanol), clarified in three changes of xylene and coverslipped with Canada balsam. The hybridized sections were viewed under a Nikon Eclipse Ni light microscope (Nikon, Tokyo, Japan) under bright-field illumination.

### Southern blotting

*Isoforms 3, 4, 5* and *6* were amplified from mouse adult cDNA using specific primers. Plasmid containing *Isoforms 1/2* was used as template in a control reaction and also to amplify the 3′-region of *Isoforms 1* to *6*. For all PCR products, an aliquot of 5 μl was electrophoresed on 1.2% agarose gel with ethidium bromide. The agarose gel was photographed and processed for Southern blotting as previously described [31]. The agarose gel was treated in 0.25 N HCl and 0.4 N NaOH. The PCR-amplified product was capillary-blotted to Nytran SuPerCharge membrane by TurboBlotter system (GE Healthcare, Amersham, UK) in a transfer solution of 20 × SSC according to the manufacturer’s instructions. The membrane was washed in 2 × SSC, and baked at 80°C for 2 hours. Then, it was rinsed in hybridization buffer comprising 5 × SSC, 2% blocking reagent (Roche Diagnostics), 0.1% sarcosine, 0.02% SDS and 50% formamide. The membrane was subjected to hybridization with Probe-GAD1 – against 3′-region of *Isoforms 1* to *6* (1 μg/ml) in the hybridization buffer overnight at 55°C. After hybridization, the membrane was washed with 2 ×, 0.5 × SSC at room temperature, 0.1 × SSC at 55°C for 30 minutes each. Then, the membrane was blocked with 1% blocking reagent in DIG buffer 1 for 30 minutes. It was incubated with alkaline phosphatase-labelled anti-digoxigenin antibody (Roche Diagnostics; 1:500 dilution) for 30 minutes at room temperature and subsequently washed twice in DIG buffer 1 for 10 minutes. For alkaline phosphatase enzyme histochemistry, the membrane was incubated with the reaction mixture containing 450 μg/ml NBT and 175 μg/ml of BCIP in DIG buffer 2 for 30 minutes at room temperature.

## Results and discussion

### GAD1 gene and mRNA splicing isoforms

**Figure 2:**
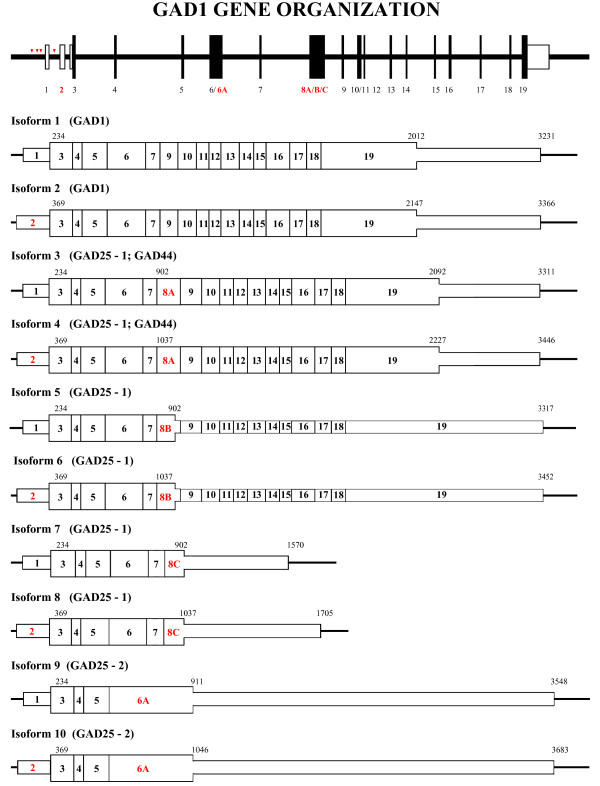
**Exon/intron structure and alternative mRNA transcripts of mouse GAD1 gene.** The new arrangement of mouse GAD1 exons and introns as determined after the analysis of genomic and cDNA sequence data using bioinformatics, 3′-RACE, RT-PCR, cloning and sequencing. Exons are shown as numbered boxes (red numbers represent alternatively spliced exons) and introns as lines. Large boxes indicate the coding DNA sequence and the small boxes the 5′- and 3′-untranslated regions. The red arrowheads are showing the locations of the alternative promoters. The schematic representation of GAD1 splicing isoforms in relation to the gene is shown below the gene structure. The length in base pairs and the position of start and stop codons are indicated above each isoform. Diagrams are not to scale.

The coding region of mouse GAD1 gene comprises 19 exons spanning more than 45 kb of genomic DNA. GAD1 gene is transcribed from multiple promoters positioned upstream of distinct 5′-noncoding exons. Initially Szabo et al. [12] described three putative promoter regions (P1, P2 and P3) just upstream of the first noncoding exon. Later, Yanagawa et al. [13] revealed the existence of the fourth liable promoter (P4) between the first noncoding exon and the first coding exon of GAD1 gene which results in the transcription of second noncoding exon. Preferential utilization of the different promoters and alternative splicing of GAD1 coding and noncoding exons result in a heterogeneous population of GAD1 mRNAs. Here we present a general scheme of GAD1 gene structure and unified numbering of the noncoding and coding exons. We also describe the existence of new coding exons and characterize new alternatively spliced mRNA isoforms (Figure 2).

*In silico* analysis of mouse GAD1 gene structure, performed in the present study, revealed two mRNA sequences (GenBank: AK047521, AK054554) in the corresponding databases that aligned to the location of potential novel GAD1 exons. Identification of the mouse GAD1 transcripts containing novel exons by RT-PCR, followed by cloning and sequencing confirmed the bioinformatics data. Here we describe the generation of ten mRNA splicing isoforms by the combined action of utilizing different promoters and alternative splicing of the coding exons.

**Figure 3:**
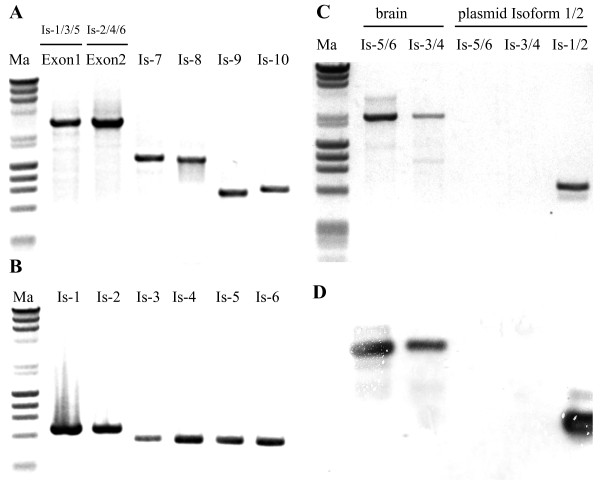
**Expression analysis of mouse GAD1 splicing isoforms in adult brain. (A)** RT-PCR analysis of the expression of GAD1 mRNA splicing isoforms in adult mouse brain by using a forward primer either in Exon1 or Exon2 and a specific reverse primer for each transcript. **(B)** Analysis of the expression of *Isoforms 1* to *6* by using the PCR product of lanes Exon1 and Exon2 in **(A)** as template for nested PCR with specific primers for each isoform. **(C)** Gel electrophoresis of *Isoforms 3/4* and *5/6*, amplified from mouse brain cDNA using specific forward primer and reverse primer, close to the position of the polyadenylation signal. Plasmid containing *Isoforms 1/2* was used as a negative control with the primers for *Isoforms 3/4* and *5/6* and as a positive control with primers amplifying the 3′-end of *Isoforms 1/2*. **(D)** Southern blotting of the gel in **(C)**. The membrane was probed with Probe-GAD1 against 3′-region of *Isoforms 1* and *2*. Ma, marker λ/HindIII-ϕX174/HaeIII.

*Isoform 1* corresponds to the reference mRNA sequence (GenBank: NM_008077) of GAD1, producing the enzymatically active full length protein GAD67. In this isoform, Exon1 is alternatively spliced to the common 3′-coding exon (Exon3). Exon1 together with 64 bp of Exon3 comprise the whole 5′-untranslated region of *Isoform 1*. Exon19 specifies the carboxyl terminal of the protein and also the entire 3′-untranslated region (Figure 2). Two overlapping polyadenylation signals (AATAAAATAAA) are located 1191 bp downstream of the TAA translation stop codon [12–14]. *Isoform 2* shares similar structure with *Isoform 1* except that it utilizes alternative promoter (P4) and has Exon2 spliced in front of Exon3 (it lacks Exon1). The translation initiation codon, stop codon and the polyadenylation signal are identical to *Isoform 1*, thus the same protein is produced. The presence of these two isoforms in adult mouse brain was confirmed by nested PCR analysis. The first PCR was performed using common forward primers in either Exon1 or Exon2 and a reverse primer at the 3′-end of *Isoforms 1* and *2* (Figure 3A). The product of the first PCR was diluted 500 times and used as a template in a second PCR reaction with specific primers only for *Isoforms 1* and *2* (Figure 3B). Using specific primers in Exon1 or Exon2 in combination with a primer close to the polyadenylation sequence the isoforms were cloned and their sequences verified by sequencing.

*Isoform 3* contains Exon1 and Exon8A which is 80 bp long (Figure 2). This exon was first described in embryonic rat brain [20] and then its existence was confirmed in the mouse [19]. *Isoform 4* shares the same structure except that it has Exon2 and is produced by utilizing the alternative promoter P4. Exon8A contains an overlapping stop/start codon (TGATG) which divides the open reading frame (ORF) of GAD1 in two overlapping ORFs. The first is coding for a 25 kDa leading peptide (GAD25-1) and the second one for a 44 kDa enzymatically active peptide (GAD44). To precisely localize the polyadenylation signal of these two isoforms, 3′-RACE was employed (data not shown). It revealed that these variants are utilizing the same polyadenylation signal as *Isoforms 1* and *2*, at least in adult mouse brain. This is in conflict with the previously described length of about 2000 bp of these transcripts detected on Northern blots [12]. To further verify the position of the polyadenylation signal and the length of these isoforms, they were amplified from adult mouse brain by specific forward primer and reverse primer in the vicinity of the polyadenylation signal of *Isoforms 1* and *2*. Plasmid containing *Isoforms 1* and *2* was used as negative control to check for the co-amplification of this variants due to the great sequence similarity; and also as a positive control to amplify the 3′-end of *Isoforms 1* and *2* (Figure 3C). Single band with the expected length was detected in the brain and no co-amplification was visible. Then the bands were blotted on a nylon membrane and probed with Probe-GAD1 against 3′-region of *Isoforms 1* and *2*. The band of *Isoforms 3* and *4* was clearly labelled together with the control band (Figure 3D). The presence of *Isoforms 3* and *4* in adult mouse brain was also confirmed by nested PCR analysis (Figure 3A and B) and the fragments were cloned and the exact sequence verified by sequencing. These results combined with the 3′-RACE data confirmed that *Isoforms 3* and *4* were utilizing the same polyadenylation signal as *Isoforms 1* and *2* in adult mouse brain.

*Isoform 5* contains Exon1 and Exon8B which is 86 bp long (Figure 2). It differs from Exon8A by the presence of 6-bp insert at the 3′-end. *Isoform 6* shares the same structure except that it has Exon2. This insert contains a downstream TGA translation stop codon that interrupts the ORF of GAD44 initiating at the overlapping TGATG codon. Thus *Isoforms 5* and *6* code only for the shorter GAD25-1 protein. To find out whether these isoforms share the same polyadenylation signal as *Isoforms 1, 2, 3* and *4* we performed 3′-RACE analysis (data not shown) and also PCR analysis identical to the one described for the previous two isoforms. PCR amplification revealed single band with the expected size and no co-amplification with *Isoforms 1* and *2*. Moreover, after the Southern blot hybridization the probe specific for the 3′-region of *Isoforms 1* and *2* clearly labelled the band of *Isoforms 5* and *6* (Figure 3C and D). The fragments were also cloned and sequenced to verify the correct sequence. The expression of these isoforms in adult mouse brain was detected by nested PCR using specific primers (Figure 3A and B).

*Isoform 8* contains Exon2, Exon8C and corresponds to a sequence in the GenBank database (GenBank: AK047521). This sequence is 1705 bp long, the translation initiation site is the same as in previous isoforms and the stop codon is located 666 bp downstream of the ATG codon in Exon8C. Exon8C is identical to the Exon8B until its 3′-splice site and then becomes homologous to the genomic GAD1 sequence (GenBank: NC_000068.7). Then it encodes two overlapping polyadenylation signals (AATAAAATAAA) located 647 bp downstream of the TGA translation stop codon. This variant was amplified from adult mouse brain cDNA, cloned and sequenced (Figure 3A). To identify the predicted splice variant containing Exon1 instead of Exon2, PCR was employed using primers listed in Figure 1. Transcript with a length of 1570 bp, which will be referred as *Isoform 7* (Figure 2), was isolated (Figure 3A), cloned and its sequence verified by sequencing and deposited into GenBank (GenBank: KM102984). These isoforms are coding for the shorter GAD25-1 leading peptide. Similar short transcripts were identified in human adult and fetal pancreatic islet cells, testis and adrenal gland [22, 23, 32]. The length of these two isoforms is almost identical to the length of the mRNA transcripts detected by Szabo et al. [19] on Northern blots. They assumed that the short length of the transcripts (corresponding to *Isoforms 3, 4, 5* and *6*) is due to the utilization of unconventional putative polyadenylation signal (AATAAT) 171 bp downstream from the translation termination codon [12]. According to our data probably these short mRNAs are corresponding to the *Isoforms 7* and *8*. Moreover, these two isoforms appear to be also more abundantly expressed than *Isoforms 3, 4, 5* and *6*, at least in adult mouse brain. Utilization of different polyadenylation signals in embryonic versus adult mouse brain for the same isoforms might be another feasible explanation for this discrepancy.

*Isoform 9* contains Exon1 and Exon6A (Figure 2), and with the exclusion of few sequencing inaccuracies (at position 1616 CT is present; at position 1647 there are two unnecessary CC bases) it corresponds to a cDNA sequence in the GenBank database (GenBank: AK054554). We have identified another isoform, referred here as *Isoform 10* (Figures 2 and 3A) by PCR, cloning and sequencing. Its sequence was deposited to GenBank (GenBank: KM102985). This isoform shares the same sequence as *Isoform 9*, except the utilization of different promoter and the presence of Exon2. The TGA termination codon is located in Exon6A, 675 bp downstream from translation initiation codon. In the so far cloned and confirmed sequences of *Isoforms 9* and *10* we were not able to identify possible polyadenylation signals, and the 3′-RACE produced inconclusive results (data not shown).

**Figure 4:**
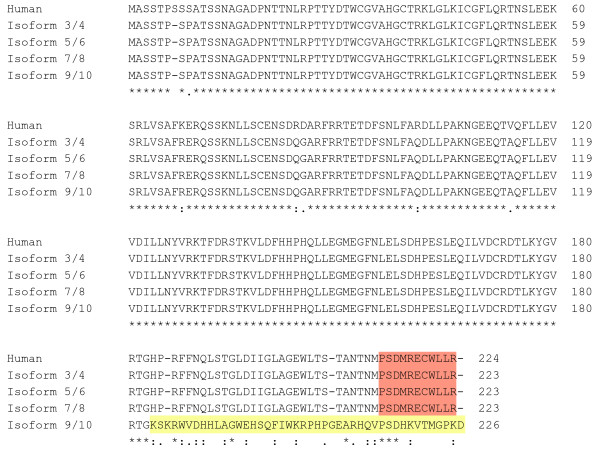
**The alignment of amino acid sequences of human and mouse GAD25.** GAD25-1 protein of 223 amino acids is produced by *Isoforms 3* to *8*. GAD25-2 protein of 226 amino acids is produce by *Isoforms 9* and *10*. The last 11 amino acids (marked in orange) of GAD25-1 and 44 amino acids (marked in yellow) of GAD25-2 are unique to these proteins. * (asterisk) indicates positions which have a single, fully conserved residue; : (colon) indicates conservation between amino acids of strongly similar properties; . (period) indicates conservation between amino acids of weakly similar properties.

*Isoforms 3* to *10* are encoding the translation of the protein GAD25 corresponding to the putative regulatory region of the full length GAD1. *Isoforms 3* to *8* share the same ORF and are producing identical GAD25-1 protein of 223 amino acids. *Isoforms 9* and *10* have ORFs that differ at the 3′-end and probably are translated into GAD25-2 protein of 226 amino acids. The last 11 amino acids of GAD25-1 and 44 amino acids of GAD25-2 are unique to these proteins, because they are derived from the alternatively spliced exons. Of note is that, the carboxyl terminal 11 amino acids (where GAD25-1 diverges from GAD1) are identical in human [22] (GenBank: NP_038473) and mouse (Figure 4).

Alternative promoters, splicing and polyadenylation of genes during embryogenesis and adulthood can lead to the synthesis of new isoforms derived from one gene. These lead to the diversity of gene expression and in the translated proteins. Our data reveal that *Isoforms 3* to *10* are predicted to be translated into truncated GAD25 proteins. The functional significance of these GAD25 proteins in normal embryonic and adult brains, and in pathophysiological conditions is still unclear. It might be implied that the generation of these transcripts is more likely to regulate GAD1 gene expression and the level of GAD activity [18, 24, 25, 33].

### Expression of GAD1 isoforms and GAD2 in different mouse tissues

**Figure 5:**
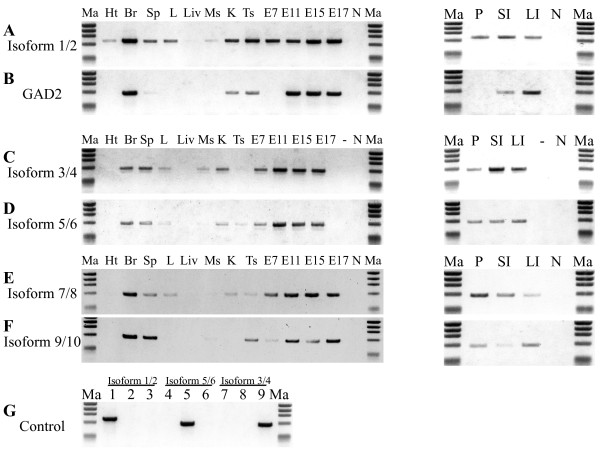
**Expression of GAD1 mRNA splicing isoforms and GAD2 in different mouse tissues and during development.** The expression level of *Isoforms 1/2* **(A)**, GAD2 **(B)**, *Isoforms 3/4* **(C)**, *Isoforms 5/6* **(D)**, *Isoforms 7/8* **(E)**, *Isoforms 9/10* **(F)** are compared by PCR amplification using mouse Multiple Tissue cDNA Panel I and cDNA from pancreas, small intestine, and large intestine as cDNA template. **(G)** Control PCR reaction to verify the specificity of the primers for *Isoforms 1/2, 3/4 and 5/6*. In the control reaction each primer pair was tested with a plasmid containing each full length insert as a template. The template in lanes 1, 4 and 7 was plasmid containing *Isoforms 1/2* as template; lanes 2, 5 and 8, plasmid containing *Isoforms 5/6*; and lanes 3, 6, 9 plasmid containing *Isoforms 3/4*. Lanes (Ht) heart; (Br) brain; (Sp) spleen; (L) lung; (Li) liver; (Ms) muscle; (K) kidney; (Ts) testis; (E7) 7-day embryo; (E11) 11-day embryo; (E15) 15-day embryo; (E17) 17-day embryo; (P) pancreas; (SI) small intestine; (LI) large intestine; (N) no template control; (−) plasmid containing *Isoforms 1/2* is used as template for the amplification; and Ma, marker ϕX174/HaeIII.

Using Mouse MTC Panel I as cDNA template and cDNAs from pancreas, small intestine and large intestine we examined the expression levels of different GAD1 mRNA isoforms and GAD2 (Figure 5). The different primer sets and specific PCR conditions for each isoform are listed in Figure 1. *Isoforms 1, 2, 3, 4, 5* and *6* share great sequence similarities. Control PCR reaction was performed to adjust the appropriate conditions for each primer pair and to verify their specificity. In the control reaction primer pairs for *Isoforms 1/2*, *3/4* and *5/6* were tested with plasmids containing each full length insert as a template. Under the specified conditions each primer pair was able to amplify only the dedicated isoform without any co-amplification (Figure 5G).

*Isoforms 1/2* were detectable at high level in brain, kidney, testis and during all embryonic days; low level of expression was detectable in spleen, lung, pancreas, small intestine, large intestine and the lowest level in heart and skeletal muscle (Figure 5A). The tissue pattern of expression of *Isoforms 3/4, 5/6, 7/8* and *9/10* was similar to that of the *Isoforms 1/2* (Figure 5C-F). The highest expression of these variants was during all embryonic days included in the MTC panel I and in the adult brain; low expression level was detected in spleen, pancreas, small intestine and large intestine; and no expression was detected in the heart. *Isoforms 3/4* were not detectable in testis; *Isoforms 5/6* were not detectable in skeletal muscle; and *Isoforms 9/10* could not be detected in lung and kidney. GAD2 in contrast was highly expressed only in brain, large intestine and during 11th, 15th and 17th embryonic days; low expression was detected in kidney, testis and small intestine (Figure 5B).

The expression pattern of *Isoforms 1/2* in the brain and non-neural tissues is similar to the previously reported results [34, 35]. GAD1 but not GAD2 is highly expressed in pancreatic islet cell of the mouse [36–38]. In contrast human and monkey islet cells produce only GAD2. Also in accordance with previous data [12, 20, 21, 33] GAD1 isoforms were moderately expressed even at the 7th embryonic day, while GAD2 became detectable later, at the 11th embryonic day.

### Quantitative RT-PCR analysis of the expression of GAD1 isoforms and GAD2 in different areas of adult mouse brain

**Figure 6:**
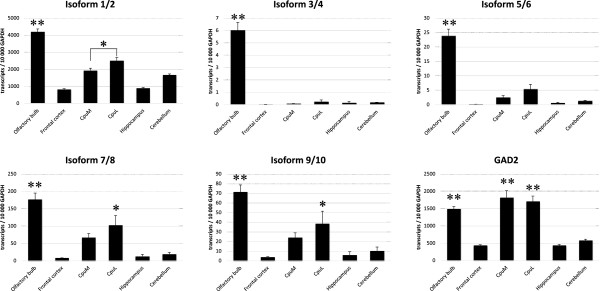
**Expression of GAD1 mRNA splicing isoforms and GAD2 in olfactory bulb, frontal cortex, medial striatum, lateral striatum, hippocampus and cerebellum of adult mouse brain.** Samples were examined for GAD transcripts by quantitative RT-PCR. Number of transcripts were calculated per 10000 transcripts of GAPDH. Data is presented as mean ± SEM of five individual animals and analyzed by one-way ANOVA followed by Tukey’s post hoc test. ** *P* ≤ 0.0001, * *P* < 0.05. (CpuM) medial striatum; (CpuL) lateral striatum.

The present study shows the expression level and expression pattern of GAD1 mRNA splicing isoforms and GAD2 in defined regions of the adult mouse brain. Expression of GAD2 was similar in frontal cortex, hippocampus and cerebellum. The highest level of expression was in the striatum followed by the olfactory bulb. The expression in medial striatum was significantly higher compared to all studied regions except olfactory bulb and lateral striatum (Figure 6; Additional file 2). These findings are in correlation with our previous results from *in situ* hybridization study of the expression of GAD1 and GAD2 in adult mouse striatum [9].

Expression of *Isoforms 1* and *2* encoding the full length GAD1 enzyme and referred in the literature as adult isoforms was highest in olfactory bulb, followed by the striatum. In the lateral striatum the expression of *Isoforms 1/2* was 1.3 folds higher than in the medial striatum. This is similar to the results from our previous study [9] showing the difference in the expression level of GAD1 in lateral versus medial striatum. In this *in situ* hybridization study we found about 1.5 folds difference in the relative expression levels of GAD1 in the lateral compared to the medial striatum. Level of expression in frontal cortex and hippocampus was almost equal and the lowest compared to the other examined regions (Figure 6; Additional file 2).

When comparing the adult GAD1 message with GAD2 we found that *Isoforms 1/2* are the predominant isoforms in all studied regions except the medial striatum where no significant difference in the expression level was observed (Figure 6; Additional file 2). In accordance to our results, *in situ* hybridization and immunohistochemical studies in adult rats clearly showed a stronger labelling for GAD1 compared to GAD2 in most of the studied regions [3, 6–8]. In contrast, Popp et al. [39] found that in the rat GAD2 mRNA was predominant in the hippocampus, and in all other regions there were not any prevalence of one of the adult GAD forms, but probably this discrepancy comes from different species that were used.

*Isoforms 3/4* and *5/6*, that are referred in the literature as embryonic isoforms, because of their high expression during early embryogenesis [1, 19–21], were barely detectable in frontal cortex, hippocampus and cerebellum. The olfactory bulb showed the highest transcript level followed by the striatum. There were no significant differences in the expression level of these isoforms between the lateral and medial striatum (Figure 6; Additional file 2). Comparison between the expression of *Isoforms 3/4* and *5/6* revealed that in the olfactory bulb there were about 4 times more *Isoforms 5/6* than *Isoforms 3/4*. In cerebellum the ratio was 7 times higher in favor of *Isoforms 5/6*. The prevalence of *Isoforms 5/6* coincides with a previous quantitative RT-PCR study in the rat [39]. In contrast Szabo et al. [19] reported that *Isoforms 5/6* were only expressed in early developmental stages (E10.5-E11.5) and were not detectable in adult brain, whereas *Isoforms 3/4* were more abundant in later embryonic stages and low level is detectable in adult brain. Probably the higher sensitivity of the quantitative RT-PCR compared to the RT-PCR is responsible for this discrepancy, but our RT-PCR data also implicates higher level of expression of *Isoforms 5/6* in adult brain (Figure 5C and D).

Predominant expression of *Isoforms 7/8* and *9/10* was in the olfactory bulb, similar to the previously described *Isoforms 3/4* and *5/6*. They showed similar low level of expression in frontal cortex, hippocampus and cerebellum. The expression in the striatum was moderate and there was not statistically significant difference between the levels in lateral and medial striatum (Figure 6; Additional file 2). Of all embryonic GAD1 mRNAs, *Isoforms 7/8* appear to be the most abundant forms in adult mouse brain. They are significantly higher than *Isoforms 3/4, 5/6* and *9/10* in olfactory bulb, frontal cortex and medial striatum; and additionally in cerebellum compared to *Isoforms 9/10*.

**Figure 7:**
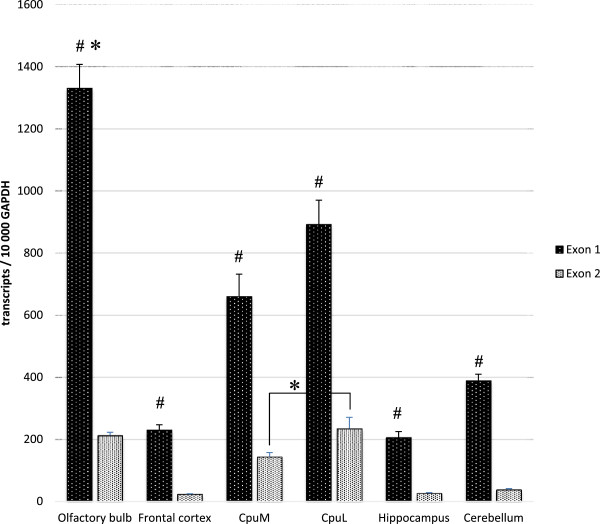
**Expression of Exon1 and Exon2 in olfactory bulb, frontal cortex, medial striatum, lateral striatum, hippocampus and cerebellum of adult mouse brain.** Samples were examined for Exon1 and Exon2 transcripts by quantitative RT-PCR. Number of transcripts were calculated per 10000 transcripts of GAPDH. Data are presented as mean ± SEM of five individual animals. Expression of Exon1 and Exon2 in each of the investigated brain regions was evaluated by Mann–Whitney *U* test. # *P* < 0.05. Comparison of the expression of Exon1 and Exon2 between the various mouse brain regions was analyzed by one-way ANOVA followed by Tukey’s post hoc test. * *P* < 0.05. (CpuM) medial striatum; (CpuL) lateral striatum.

Expression of the two alternatively spliced 5′-noncoding exons was analyzed in the studied brain regions in an attempt to investigate the level of activity of the different promoters (Figure 7; Additional file 2). Expression pattern of the two transcripts was similar to that of *Isoforms 1/2*. The number of transcripts was similar in frontal cortex, hippocampus and cerebellum. The highest level of expression was in olfactory bulb, followed by the striatum. Significant difference in the expression in lateral striatum compered to medial striatum was demonstrated only for Exon2. Comparing the expressions of each exon revealed that Exon1 is the predominant form in all regions. These results closely resemble the findings of Yanagawa et al. [13] that have found by RT-PCR the abundance of Exon1 in the mouse brain. It might be concluded that promoter P1, that resembles the promoters of housekeeping genes and minor promoters P2 and P3 [12] are preferentially activated in the adult mouse brain.

### *In situ*hybridization for GAD1 *Isoforms 7/8*and *Isoforms 9/10*in adult mouse brain

**Figure 8:**
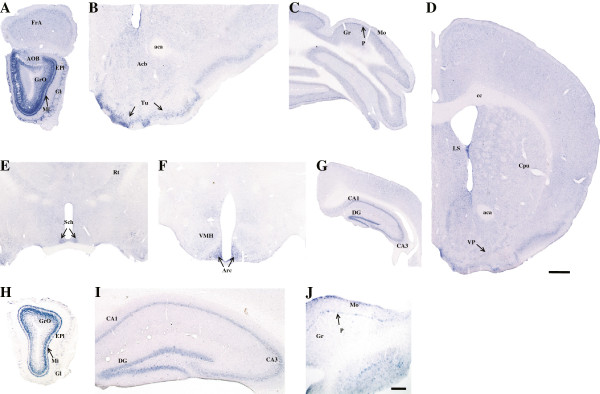
**Expression of GAD1** ***Isoforms 7/8*** **and** ***Isoforms 9/10*** **in adult mouse brain detected by** ***in situ*** **hybridization.** Expression of *Isoforms 7/8* **(A-G)** and expression of *Isoforms 9/10* **(H-J)**. Moderate expression of *Isoforms 7/8* was detected in olfactory bulb **(A)**. Low expression was detectable in olfactory tubercle and accumbens nucleus **(B)**; cerebellum **(C)**; lateral septal nucleus, ventral pallidum and striatum **(D)**; suprachiasmatic nucleus **(E)**; VMH and arcuate nucleus **(F)**; hippocampus **(G)**. Expression of *Isoforms 9/10* could be detected only in olfactory bulb **(H)**, hippocampus **(I)** and cerebellum **(J)**. Acb, accumbens nucleus; aca, anterior commissure; AOB, accessory olfactory bulb; Arc, arcuate nucleus; Cpu, caudate putamen (striatum); cc, corpus callosum; DG, dentate gyrus; EPl, external plexiform layer of olfactory bulb; FrA, frontal association cortex; Gl, glomerular layer of olfactory bulb; GrO, granular cell layer of olfactory bulb; Gr, granular cell layer of cerebellum; LS, lateral septal nucleus; Mi, mitral cell layer of olfactory bulb; Mo, molecular cell layer of the cerebellum; P, Purkinje cells; Rt, reticular nucleus; Sch, suprachiasmatic nucleus; Tu, olfactory tubercle; VMH, ventromedial hypothalamic nucleus; VP, ventral pallidum. Scale bar = 100 μm **(J)**, 200 μm **(B, C, E, F** **and I)**, 300 μm **(A, G** **and H)**. Scale bar = 1 mm **(D)**.

Using specific digoxigenin-labelled riboprobes located in the unique 3′-noncoding region of *Isoforms 7/8* and *9/10* we investigated their expression in the adult mouse brain. Because of the enormous sequence homology between the other GAD1 isoforms it is impossible to prepare digoxigenin-labelled riboprobes with enough specificity. The level of expression of Isoforms *7/8* and *9/10* was low, but resembled closely the expression of GAD1 as reported in the rat and mice [6–9]. The highest expression of *Isoforms 7/8,* revealed by quantitative RT-PCR, in olfactory bulb could be confirmed by non-radioactive *in situ* hybridization (Figure 8A). The cells of the granule cell layer were intensely labelled and moderate labelling was detectable in the glomerular cell layer and in the accessory olfactory bulb. In frontal cortex and other cortical areas there was moderate expression of *Isoforms 7/8* in numerous inhibitory interneurons. Strong labelling could be seen in the cell of olfactory tubercle, while the staining in the ventral pallidum was moderate (Figure 8B and D). Low expression could be demonstrated in the striatum and accumbens nucleus (Figure 8B and D). In the diencephalon, strong labelling was visible in the suprachiasmatic nucleus (Figure 8E) and arcuate nucleus (Figure 8F), while in the ventromedial hypothalamus (Figure 8F) and the reticular nucleus of the thalamus (Figure 8E) it was weaker. In the hippocampus many inhibitory interneurons were strongly labelled for *Isoforms 7/8* (Figure 8G). The most intense labelling was in the granule cells of the dentate gyrus, followed by the cells in the pyramidal layer of CA3 region and some cells in the hilar region. GAD1 mRNA and immunoreactivity have been described in the granule cells and their mossy fibers which represent the major glutamatergic pathway of the hippocampus. Markedly increased expression of adult GAD1 and so called embryonic GAD1 variants (*Isoforms 1* to *6*) was demonstrated in adult models of epilepsy by *in situ* hybridization, immunohistochemistry and RT-PCR [40, 41]. Using radioactive *in situ* hybridization with an oligonucleotide probe Szabo et al. [12] detected trace amounts of embryonic GAD1 variants in the granule cells of rat dentate gyrus, which was markedly enhanced after kainic acid induced seizures. The probe used in that experiment was specific to Exon8 and could label *Isoforms 3, 4, 5, 6, 7* and *8* described here. Specific labelling for *Isoforms 7/8*, distinguishable from the background, could not be detected in the brainstem. In the cerebellum strong labelling was visible in the Purkinje cells, and moderate to low labelling in the molecular and granular cell layers (Figure 8C). Radioactive *in situ* hybridization studies using oligonucleotide probe in the vicinity of Exon8 (recognizing *Isoforms 3/4, 5/6* and *7/8*) have described sequential expression of embryonic and adult GAD1 transcripts [21, 39]. In areas like olfactory bulb with high level of cell proliferation and migration embryonic variants are more abundant.

The level of expression of *Isoforms 9/10* was significantly lower than *Isoforms 7/8*. Strong labelling was seen in the olfactory bulb (Figure 8H). Also, some interneurons of the dentate gyrus, CA and the hilus of the hippocampus were moderately labelled (Figure 8I). In the cerebellum the labelling was intense in the Purkinje cells, and very low in the cells of the molecular and granular cell layers (Figure 8J).

## Conclusions

In summary, GAD1 gene in mouse brain is extensively regulated at the level of transcription. So far ten alternatively spliced isoforms were identified, which are produced by combined action of different promoter utilization and splicing of coding exons. *Isoform 1* and *Isoform 2* are translated into the full length, enzymatically active GAD1 protein with 67 kDa molecular weight. *Isoforms 3* to *10* are responsible for the production of a short, enzymatically inactive peptide with unknown function and molecular weight of 25 kDa (GAD25-1 and GAD25-2). GAD25 is highly conserved between human and mouse. *Isoforms 3* and *4* are capa-ble of producing a second 44 kDa peptide with enzyme activity (GAD44). All alternatively spliced isoforms share similar pattern of expression in different mouse tissues. The highest level of expression in the brain is in the olfactory bulb, followed by the striatum. The most abundant isoforms in the adult brain are *Isoforms 1* and *2*. The isoforms producing GAD25 are barely detectable in the adult mouse brain with the exception of the comparatively high expression in the olfactory bulb. This predominant localization in the olfactory bulb was confirmed for *Isoforms 7/8* and *9/10* by *in situ* hybri-dization. The low expression of the isoforms responsible for the synthesis of GAD25 implies its limited relevance in postnatal development, but their significantly higher expression in areas associated with neurogenesis and high synaptic plasticity like olfactory bulb and hippocampus, suggests their functional role in the proliferation and maturation of neuroblasts, synaptogenesis and synaptic plasticity. The detailed description of GAD1 splicing isoforms could be useful for further studies on the regulation of GAD1 expression during nervous system development and plasticity, and also during certain pathological conditions.

## Electronic supplementary material

Additional file 1: **Sequence of the standards used for the quantitative RT-PCR.** (PDF 75 KB)

Additional file 2: **Statistical analysis of the data from quantitative RT-PCR experiments.** (PDF 168 KB)
