# Supplementary material for: Glutamic acid decarboxylase 1 alternative splicing isoforms: characterization, expression and quantification in the mouse brain
Source: BMC Neurosci. 2014 Oct 16;15:114. doi: 10.1186/1471-2202-15-114 (PMC4295415; doi:10.1186/1471-2202-15-114)
Supplement: Supplementary file 1 — Additional file 1: Sequence of the standards used for the quantitative RT-PCR. (PDF 75 KB) [file 12868_2014_3802_MOESM1_ESM.pdf]

Supplement 1. Sequence of the standards used for the quantitative RT-PCR.

Standard-1

Exon 1 → Exon 2 → *Isoform 3/4* → GAPDH

tttCTTCTTCAGGCTCTCCCGTGCcggaccagggatcgtgcaagcaaggaagcagccctggggtgacacccagcacgtactc
ctgtgacagagccgagcccagcccagccccgggacgcttcgcagaggagtcgcgggagggtccagctcgctgtcgctgaaccgagcctgttc
ctgcgcccagtctgcgggggacccttgaaccGTAGAGACCCCAAGACCACCGAGtttttTCTCCTTTTACCCT
CTGCCAccttctctggctcagtgaaacgaactctggggactaggtagagtagaaacagtgaatctgggacggagcctcgctctagggttga
gaaagctagAAACGAGGTGGGACAAGAAACTtttttAGTGTGACCTCCAGAGGTTCacatatgaaattgca
cccgtgtttgttctcatggaacagattactcttaagaagatgagagagatcgttggatggtcaaataaagatggtgatgggatattttCTCCTGG
GGGAGCCATATCCAtttttGCATGGCCTTCCGTGTTCctacccccaatgtgtccgtcgtggatctgacgtgccgcctg
gagaaacctgccaagtatgatgacaTCAAGAAGGTGGTGAAGCAGGttt

Standard-2

Isoform 1/2 → GAD2 → GAPDH

tttTCGATTTTTCAACCAGCTCTCTACTggtttggatatcattggtttagctggtgaatggctgacatcgactgccaataccA
ATATGTTCACATATGAAATTGCACtttttCATTGATAAGTGTTTGGAGCTAGCAgagtatttatacact
atcataaaaaaccgagaaggctatgaaatggtgtttgatgggaagcctcaacacacaaatgtctgcttctggtTTGTACCTCCTAGTT
TGCGCACtttttGCATGGCCTTCCGTGTTCctacccccaatgtgtccgtcgtggatctgacgtgccgcctggagaaacctgc
caagtatgatgacaTCAAGAAGGTGGTGAAGCAGGttt

Standard-3

Isoform 7/8 → *Isoform 9/10* → *Isoform 5/6* → GAPDH

tttCACTTGGCTGGGTCGGTCTAggcatctctttccagctaggatggtcttggctcagcctacagacaccaaagcttaagaacttc
aaggcaaagcaagaaaaagagccttagcgtttagtaAACGGATTCTGTCTTTAGGCTCAtttttTGACTTTCTCCC
CTTACTTGACATTaaaccatgtattactcagcatcatgtctgtgagaactaaaagcaaatctgggtttgggcctaattgtacctgcctgtg
aagccaacacttAGAAAATGAGGGTGAGATCAAGAAGtttttTGACCTCCAGAGGTGATGGTtcacata
tgaaattgcacccgtgtttgttctcatggaacagattactcttaagaagatgagagagatcgttggatggtcaaataaagatggtgatgggatattttC
TCCTGGGGGAGCCATATCCAtttttGCATGGCCTTCCGTGTTCctacccccaatgtgtccgtcgtggatctgacg
tgccgcctggagaaacctgccaagtatgatgacaTCAAGAAGGTGGTGAAGCAGGttt
