# Supplementary material for: Glutamic acid decarboxylase 1 alternative splicing isoforms: characterization, expression and quantification in the mouse brain
Source: BMC Neurosci. 2014 Oct 16;15:114. doi: 10.1186/1471-2202-15-114 (PMC4295415; doi:10.1186/1471-2202-15-114)
Supplement: Supplementary file 2 — Additional file 2: Statistical analysis of the data from quantitative RT-PCR experiments. (PDF 168 KB) [file 12868_2014_3802_MOESM2_ESM.pdf]

Supplement 2. Statistical analysis of the data from quantitative RT-PCR experiments.

A. Comparison of the expression of each GAD1 isoform and GAD2 between the various mouse brain regions as analyzed by one-way ANOVA followed by Tukey's post hoc test. ** $P \leq 0.0001$, * $P < 0.05$, *ns* – not significant. (Olf) olfactory bulb; (Fr) frontal cortex; (CpuM) medial striatum; (CpuL) lateral striatum; (Hp) hippocampus; (Crb) cerebellum.

	Olf-Fr	Olf-CpuM	Olf-CpuL	Olf-Hp	Olf-Crb	Fr-CpuM	Fr-CpuL	Fr-Hp	Fr-Crb	CpuM-CpuL	CpuM-Hp	CpuM-Crb	CpuL-Hp	CpuL-Crb	Hp-Crb
Is 1/2	**	**	**	**	**	**	**	ns	*	*	**	ns	**	*	*
Is 3/4	**	**	**	**	**	ns	ns	ns	ns	ns	ns	ns	ns	ns	ns
Is 5/6	**	**	**	**	**	ns	ns	ns	ns	ns	ns	ns	ns	ns	ns
Is 7/8	**	*	*	**	**	ns	*	ns	ns	ns	ns	ns	*	*	ns
Is 9/10	**	*	*	**	**	ns	*	ns	ns	ns	ns	ns	*	ns	ns
GAD2	**	ns	ns	**	**	**	**	ns	ns	ns	**	**	**	**	ns

B. Comparison of the expression of each GAD1 isoform and GAD2 in one brain region as analyzed by one-way ANOVA followed by Tukey's post hoc test. ** $P \leq 0.0001$, * $P < 0.05$, *ns* – not significant. (Olf) olfactory bulb; (Fr) frontal cortex; (CpuM) medial striatum; (CpuL) lateral striatum; (Hp) hippocampus; (Crb) cerebellum.

	1/2-3/4	1/2-5/6	1/2-7/8	1/2-9/10	1/2-GAD2	3/4-5/6	3/4-7/8	3/4-9/10	3/4-GAD2	5/6-7/8	5/6-9/10	5/6-GAD2	7/8-9/10	7/8-GAD2	9/10-GAD2
Olf	**	**	*	*	*	*	*	*	*	*	*	*	*	*	*
Fr	*	*	*	*	*	ns	*	*	*	*	*	*	*	*	*
CpuM	*	*	*	*	ns	ns	*	*	*	*	*	*	*	*	*
CpuL	*	*	*	*	*	ns	ns	ns	*	ns	ns	*	ns	*	*
Hp	*	*	*	*	*	ns	ns	ns	*	ns	ns	*	ns	*	*
Crb	**	**	**	**	*	*	ns	ns	**	ns	ns	*	*	*	*

C. Expression of Exon1 and Exon2 in each of the investigated brain regions as evaluated by Mann-Whitney U test. * $P < 0.05$. (CpuM) medial striatum; (CpuL) lateral striatum.

	Olfactory bulb	Frontal cortex	CpuM	CpuL	Hippocampus	Cerebellum
Exon1-Exon2	*	*	*	*	*	*

D. Comparison of the expression of Exon1 and Exon2 between the various mouse brain regions as analyzed by one-way ANOVA followed by Tukey's post hoc test. ** $P \leq 0.0001$, * $P < 0.05$, *ns* – not significant. (Olf) olfactory bulb; (Fr) frontal cortex; (CpuM) medial striatum; (CpuL) lateral striatum; (Hp) hippocampus; (Crb) cerebellum.

	Olf-Fr	Olf-CpuM	Olf-CpuL	Olf-Hp	Olf-Crb	Fr-CpuM	Fr-CpuL	Fr-Hp	Fr-Crb	CpuM-CpuL	CpuM-Hp	CpuM-Crb	CpuL-Hp	CpuL-Crb	Hp-Crb
Exon 1	**	**	**	**	**	**	**	ns	ns	ns	**	*	**	**	ns
Exon 2	**	ns	ns	**	**	*	**	ns	ns	*	*	*	**	**	ns
